# Protective role of MG53 against ischemia/reperfusion injury on multiple organs: A narrative review

**DOI:** 10.3389/fphys.2022.1018971

**Published:** 2022-11-21

**Authors:** Bowen Xu, Chunxiao Wang, Hongping Chen, Lihui Zhang, Lei Gong, Lin Zhong, Jun Yang

**Affiliations:** ^1^ The 2nd Medical College of Binzhou Medical University, Yantai, Shandong, China; ^2^ Department of Cardiology, Yantai Yuhuangding Hospital, Yantai, Shandong, China; ^3^ Medical Department of Qingdao University, Qingdao, Shandong, China

**Keywords:** MG53, ischemia/reperfusion injury (I/R injury), multiple organs, protective, review

## Abstract

Ischemia/reperfusion (I/R) injury is a common clinical problem after coronary angioplasty, cardiopulmonary resuscitation, and organ transplantation, which can lead to cell damage and death. Mitsugumin 53 (MG53), also known as Trim72, is a conservative member of the TRIM family and is highly expressed in mouse skeletal and cardiac muscle, with minimal amounts in humans. MG53 has been proven to be involved in repairing cell membrane damage. It has a protective effect on I/R injury in multiple oxygen-dependent organs, such as the heart, brain, lung, kidney, and liver. Recombinant human MG53 also plays a unique role in I/R, sepsis, and other aspects, which is expected to provide new ideas for related treatment. This article briefly reviews the pathophysiology of I/R injury and how MG53 mitigates multi-organ I/R injury.

## Graphical Abstract

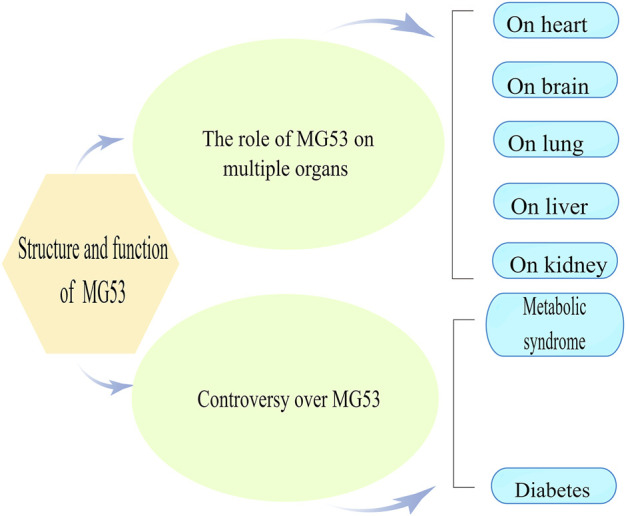

## Introduction

Mitsugumin-53 (MG53), also known as TRIM72, is a muscle-specific triple motif family protein from our immunoproteomics library (Weisleder et al., 2008). Compared with other TRIM protein family members, they are very similar in structure which contains the tripartite motif that includes a Ring finger, one zinc-binding motif called B-box, and coiled-coil (CC)moieties. As a unique protein in the TRIM family, the MG53 has a SPRY domain at the carboxy terminus (Reymond et al., 2001; Cai et al., 2009a; Park et al., 2010) and is mainly expressed in myocardium sarcolemma and skeletal muscle sarcolemma (Lee et al., 2010). Previous studies have shown that MG53 can be detected in alveolar epithelial cells and the kidney, which impacts the lungs and kidneys under physiological or pathological conditions (Table 1) (Jia et al., 2014; Duann et al., 2015). While many studies support the protective role of MG53 in cardiovascular disease (Jiang et al., 2021; Zhong et al., 2021), no reports of MG53 on its role in multi-organ Ischemia/reperfusion (I/R) injury have been published. Here, we are intended to review the role of MG53 in I/R injury.

**TABLE 1:** List of studies on the role of MG53 in multiple organs.

Tissues expression	Localization	Method for detection	Mechanisms
Skeletal muscle	Specifically expressed in the sarcolemma lipid rafts (Lee et al., 2010)	Western blotting and Immunofluorescence	Facilitate cell membrane repair (Cai et al., 2009a)
Heart	Specifically expressed in the sarcolemma lipid rafts (Lee et al., 2010)	Western blotting and Immunofluorescence	Facilitate cell membrane repair (Wang et al., 2010)
			Activate PI3K-Akt-GSK3β pathway (Cao et al., 2010) and RISK pathway (Zhang et al., 2011)
Brain	Not Detected	Not applicable	Reduce apoptosis, inhibit the release of inflammatory cytokines and mitochondrial dysfunction (Gharibani et al., 2015; Ma et al., 2020)
Lung	Type 1 and 2 alveolar epithelial cells (Jia et al., 2014)	Western blotting and Immunohistochemical staining	Facilitate cell membrane repair (Jia et al., 2014)
Liver	Not Detected	Not applicable	Combined action with dysferlin reduces HIRI-induced hepatocyte membrane damage (Matsuda et al., 2012)
Kidney	Proximal tubular epithelium (Duann et al., 2015)	Quantitative immunoblotting	Facilitate cell membrane repair (Duann et al., 2015)

### I/R injury

Ischemia is a lack of blood supply to tissue due to obstructed arterial flow, resulting in a shortage of oxygen and nutrients required for cell metabolism. Ischemic injury leads to cellular dysfunction, damage, or death, a process largely dependent on the degree and duration of blood supply disruption. Different organs have different susceptibilities to ischemic injury. After a few minutes of hypoxia in the brain, brain cells begin to suffer irreversible damage, their function is affected, and the process is almost irreversible, while muscle tissue can withstand ischemia for 60–90 min without irreversible damage (Duehrkop and Rieben, 2014). The ideal goal to prevent tissue death after local ischemia is hematologic reconstruction and restoration of blood flow as soon as possible, the benefits of which to body tissues are not as expected. During long-term ischemia, anaerobic metabolism and the accumulation of lactic acid deplete ATP and decrease intracellular pH, leading to dysfunction of ATP enzyme transport, increased intracellular and mitochondrial calcium levels (calcium overload), and eventually cell swelling, rupture, and death (Kalogeris et al., 2012). Although reperfusion restores oxygen levels, the surge of reactive oxygen species promotes neutrophil infiltration of ischemic tissue and exacerbates ischemic injury. (Kalogeris et al., 2012; Brown and Griendling, 2015). In 1960, Jennings et al. found that reperfusion after ischemia aggravated myocardial necrosis in canines for the first time. Even if the normal or near normal coronary blood flow is restored after reperfusion, mechanical dysfunction still existed (Jennings et al., 1960). There are many theories about the molecular mechanism of reperfusion injury, and some of them are pretty powerful, but they have not been fully elucidated. Here are some mainstream views: 1. Overproduction of reactive oxygen species (ROS) 2. intracellular and mitochondrial Ca2+ overload 3. Opening of the mitochondrial permeability transition pore 4. pronounced inflammatory responses 5. endothelial dysfunction (Yellon and Hausenloy, 2007; Kalogeris et al., 2016).

## Overview of biological activities of MG53

### The role of MG53 in repairing cell membrane damage

**FIGURE 1:**
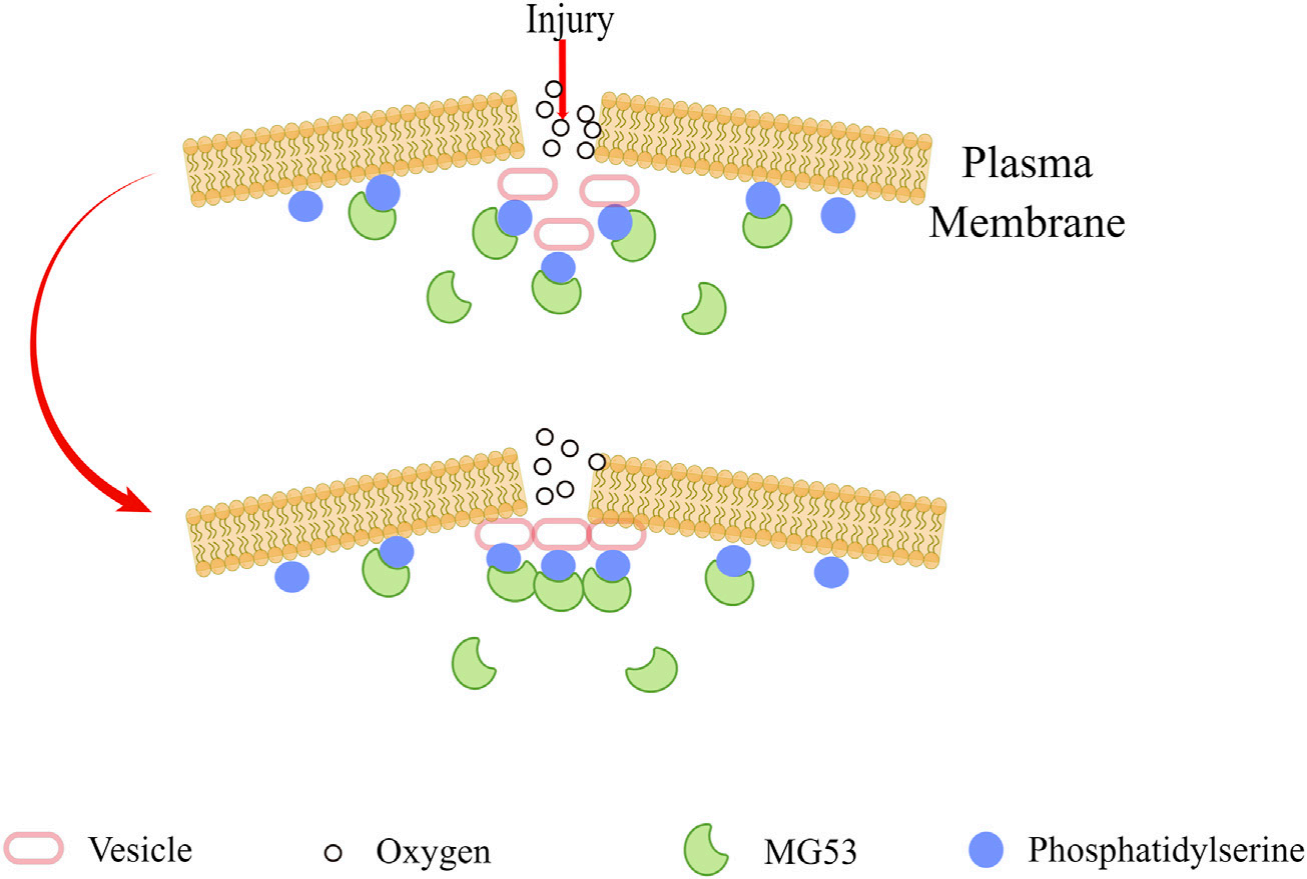
When the plasma membrane was damaged, MG53 sensed the oxidized extracellular environment, and by combining with phosphatidylserine, it adhered to the plasma membrane and intracellular vesicles, and finally gathered at the injury site to seal the damaged membrane.

The importance of the cell membrane to the organism is self-evident. It is a barrier to prevent extracellular substances from freely entering the cell, to ensure the relative stability of the intracellular environment, and to make various biochemical reactions proceed in an orderly manner (Nicolson, 2013). In order to maintain cell homeostasis, eukaryotic cells protect the integrity of their plasma membranes in some ways, such as active recycling and repair, to deal with various sources of damage (McNeil et al., 2003). Repairing plasma membrane damage is an essential aspect of normal cell physiology, and the disruption of this process can lead to many different pathophysiologies (Cooper and McNeil, 2015). Previous studies showed that MG53 is responsible for plasma membrane repair (Cai et al., 2009a). Acute destruction of the plasma membrane will cause the inside of the cell to be exposed to the external oxidative environment. The oxidation state of MG53 may signal to activate the acute membrane repair process and mediate plasma membrane resealing, in which MG53 interacts with phosphatidylserine and promotes the transport of vesicles containing phosphatidylserine to the site of membrane damage (Figure 1) (Cai et al., 2009a). Many factors regulate the membrane repair process mediated by MG53. The research of Wang et al. showed that membrane cholesterol is an indispensable molecular participant that initiates MG53 translocation in myocardial membrane repair (Wang et al., 2010). Zhu et al. found that Polymerase I and Transcript Release Factor (PTRF) can act as a docking protein of MG53 in the process of membrane repair by binding exposed cholesterol at the injury site, and cells lacking endogenous PTRF show defective transport of MG53 to the injury site (Zhu et al., 2011). MG53 protein acts as an oxidation sensor to gather cell vesicles at the site of membrane damage to form a membrane patch, and the entry of Ca^2+^promotes the fusion of vesicles and plasma membrane. Ca^2+^ is necessary for fusing vesicles and plasma membranes but not for vesicle transport (Cai et al., 2009a; Hwang et al., 2011). Cai et al. found that the interaction of MG53, dysferlin, and caveolin-3 (CaV3) is essential for the repair of acute membrane damage in striated muscle (Cai et al., 2009b). They also found that MG53 can regulate the membrane sprouting and exocytosis of muscle cells and regulate this process by interacting with Cav3. (Cai et al., 2009c). It is worth noting that combining Zn2+ with the RING and B-box motifs of MG53 is essential for assembling the cell membrane repair mechanism (Cai et al., 2015). Previous data showed that the formation of disulfide bonds on Cys242 is critical for MG53 oligomerization and the initiation of cell membrane repair (Cai et al., 2009a). Based on this, Hwang et al. found that destroying MG53 oligomerization by chemically modifying cysteine residues before membrane damage may disrupt the MG53-mediated membrane repair process. The leucine zipper motif is essential for the formation of homodimers of MG53, which regulates the redox-dependent oligomerization of MG53, and the CC domain pair also plays an equally important role (Hwang et al., 2011). There are two conserved leucine zipper motifs (LZ1 and LZ2) in the CC domain of MG 53, which are very conservative in different animal species. If LZ1 and LZ2 are mutated, the oligomerization of MG 53 will decrease, and the cell membrane repair function of MG53 will be destroyed. 3Notably, LZ one will weaken the oligomerization of MG53, while LZ2 will not (Hwang et al., 2011).

### Effect of MG53 on cardiac I/R injury

According to the latest report from WHO, the world’s biggest killer is ischemic heart disease which is responsible for 16% of the world’s total deaths. Since 2000, the most significant increase in fatalities has been for this disease, rising by more than 2 million to 8.9 million deaths in 2019 (Organization, 2020). Although cardiovascular health has improved in recent years, the global health burden of cardiovascular diseases is still high. Therefore, it is urgent to study the possible mechanisms of cardiovascular disease. Murry et al. first reported Ischemic preconditioning (IPC) in 1986. They found that multiple brief episodes of ischemic might delay cell death after coronary artery occlusion, thereby protecting the myocardium from subsequent ischemic damage (Murry et al., 1986). IPC also plays a vital role in other organs, such as the brain, liver, and kidney (Zager et al., 1985; Chen and Simon, 1997; Koti et al., 2003).

It has been confirmed that IPC and ischemic postconditioning (IPostC) can reduce myocardial injury caused by I/R (Murry et al., 1986; Zhao et al., 2002). IPC mainly involves two essential pathways: the reperfusion injury salvage kinase (RISK)pathway and the survivor activating factor enhancement (SAFE) pathway. The RISK pathway consists of PI3K-Akt-GSK3βand ERK1/2 signaling events, whereas the SAFE pathway involves activation of tumor necrosis factor-α and the JAK-STAT3 axis (Crisostomo et al., 2006; Lacerda et al., 2009; Lecour, 2009). In 2003, Zhao et al. first proposed IPostC, which is another form of cardioprotection. IPostC is achieved by shortly interrupting the perfusion cycle in the early stages of perfusion, which has been observed to save coronary artery endothelium and cardiomyocytes (Zhao et al., 2002).

With the deepening of the research, more and more proofs show that MG53 plays a vital role in IPC and IPostC (Cao et al., 2010; Zhang et al., 2011). As shown in the study of Cao et al., MG53 deficiency completely abolished IPC-mediated cardioprotection. In MG53 knockout mice, IPC failed to reduce the area of IR-induced myocardial infarction compared with wild-type mice (Cao et al., 2010). Cao et al. found that the lack of MG53 completely eliminated IPC-induced PI3K activation, indicating that MG53 is necessary for IPC-induced PI3K activation, which is a crucial component of the pro-survival RISK pathway. In contrast, MG53 does not participate in the SAFE pathway because IPC-induced STAT-3 phosphorylation remains intact in MG53-deficient hearts (Cao et al., 2010). The research by Shan et al. showed that during cardiac ischemic preconditioning, the reactive oxygen species level of myocardial cells was increased, which activated protein kinase C-δ(PKC-δ) and induced the secretion of MG53 protein (Shan et al., 2020). The phosphorylation levels of several key pro-survival kinases were significantly increased due to overexpression of MG53, including Akt, GSK3β, and ERK1/2, over their respective control (Fujio et al., 2000; Tong et al., 2002; Shiraishi et al., 2004). The study by Wang et al. found that when I/R injured the heart, MG53 could sense and promote plasma membrane translocation (Wang et al., 2010). Meanwhile, immunostaining of myocardial sections showed that MG53 accumulated in the myocardium, which indicated that the I/R activated the repair mechanism mediated by MG53. MG53 ablation aggravated myocardial damage caused by I/R, manifested by mitochondrial dysfunction and cardiomyocyte loss (Wang et al., 2010). In a recent study, Gumpper-Fedus et al. found that during ischemia-reperfusion-induced oxidative stress, MG53 binds to cardiolipin, a mitochondria-specific phospholipid, preventing damage to mitochondria, and maintains their integrity (Gumpper-Fedus et al., 2022). Wang et al. found that I/R-induced heart damage can lead to necrosis, while MG53 has a regulatory effect on RIPK1-dependent necrosis during I/R injury, which inhibits I/R damage cardiac necrosis by downregulating ubiquitination-dependent RIPK1 expression (Wang et al., 2022).

Although Lemckert et al. demonstrated that MG53 was an effective biomarker for mice heart myocardial injury and dysfunction, the expression of MG53 was extremely low or undetectable in samples of pediatric patients undergoing surgery to correct congenital structural heart defects. This research indicates that MG53 cannot be used as a clinical biomarker of human myocardial damage, nor can it be used as an endogenous cardioprotective agent for ischemic preconditioning or postprocessing (Lemckert et al., 2016). However, Guo et al. reported the expression of MG53 in the human atrium for the first time, which was verified by immunohistochemical staining, quantitative PCR, and western blotting in patients undergoing cardiac surgery (Guo et al., 2018). These differences between studies may be due to different experimental methods or different antibodies used, and further studies are needed to prove whether MG53 is expressed in the human heart. In 2020, Xie et al. used enzyme-linked immunosorbent assay (ELISA) to determine the concentration of MG53 in the serum of 639 patients undergoing coronary angiography. Logistic and linear regression was used to analyze the relationship between MG53 and coronary heart disease. They found that patients with stable cardiovascular disease and acute myocardial infarction had elevated serum MG53 levels (Xie et al., 2020a), and subsequent studies have shown that MG53 is a valuable prognostic indicator for patients with acute myocardial infarction (AMI) (Xie et al., 2020b), which can provide new ideas for clinical practice.

### Effects of MG53 on cerebral I/R injury

Stroke is an acute cerebrovascular disease, which is the leading cause of death and long-term disability. The majority of strokes are ischemic brain damage caused by occlusion of cerebral arteries (Virani et al., 2020). An ischemic stroke usually leads to lower blood oxygen levels and hypoperfusion, which in turn leads to neurotoxic events. Cerebral ischemic injury can lead to the formation of cerebral edema and increased intracranial pressure (ICP), which compresses the cerebral vessels and weakens cerebral perfusion in the ischemic area (Simard et al., 2007). Timely restoration of blood flow is necessary to prevent ischemic tissue infarction, but oxygenated blood reperfusion can lead to aggravation of brain damage, also known as I/R injury. Oxidative stress, inflammation, and apoptosis are the primary cellular responses of brain tissue after I/R injury (Fang et al., 2015; Palencia et al., 2015). Brain I/R injury increases the phosphorylation of extracellular signal-regulated kinase 1/2 (ERK1/2), induces the expression of membrane G protein-coupled receptor kinase 2 (GRK2), and reduces the expression of cortical microvascular matrix metalloproteinase-9 (MMP-9) and cytoplasmic GRK2 (Zheng et al., 2007; Sarshoori et al., 2014). Due to the blood-brain barrier (BBB), many neuroprotective agents confirmed within *in vitro* studies have lost their effect within *in vivo* experiments. In mouse tissues, MG53 is predominantly expressed in skeletal and cardiac muscles and cannot be detected in neurons (Cai et al., 2009a; Yao et al., 2016). However, Yao et al. provided evidence that I/R injury caused an abundant accumulation of MG53 in the brain tissue. (Yao et al., 2016). Moreover, their research showed that recombinant human MG53 (rhMG53) could cross the BBB to the I/R damaged brain. Intravenous administration before or after ischemia can effectively reduce brain injury, inhibit apoptosis, and activate the pro-survival RISK signaling (Yao et al., 2016). Brain I/R injury leads to the decrease of Akt phosphorylation and the inhibition of GSK3β activation, which can be restored by rhMG53 treatment (Yao et al., 2016). Some studies have shown that I/R-induced brain injury leads to the activation of caspase3, which is the core protein of the apoptotic cascade reaction pathway (Porter and Janicke, 1999; Galluzzi et al., 2012). Caspase3 activation is an essential condition for cell apoptosis, and rhMG53 treatment could effectively suppress caspase3 activation (Chen et al., 2015; Gharibani et al., 2015). MG53 could significantly inhibit the expressions of TNF-α, TLR4, NLRP3, Caspase1, and IL-1β, reduce the neuroinflammatory response, promote the proliferation and migration of human umbilical cord mesenchymal stem cells (hUC-MSCs), inhibit the release of inflammatory cytokines, and resist LPS-induced apoptosis and mitochondrial dysfunction (Guan et al., 2019; Ma et al., 2020). The combination of MG53 and hUC-MSCs promoted neurogenesis by reducing apoptosis and improving PI3K/Akt-GSK3β signaling pathway. MG53 protects hUC-MSCs from inflammatory injury by inhibiting the NLRP3/Caspase-1/IL-1β axis and synergistically enhances its efficacy on the brain damaged by neuroinflammation. RhMG53 protein has a protective effect on H2O2-induced oxidative damage of hUC-MSCs, and promotes the proliferation and migration of hUC-MSCs, reducing cerebral edema and neurological deficits (Guan et al., 2019; Ma et al., 2020).

### Effects of MG53 on lung I/R injury

Since the 1980s, with the success of lung transplantation, it has become the main treatment for most end-stage lung diseases. Although lung surgical techniques and perioperative care have improved, lung injury caused by I/R is still a significant cause of early morbidity and mortality after lung transplantation (King et al., 2000). I/R-induced lung injury can occur in acute pulmonary embolism, heart organ transplantation, and cardiopulmonary bypass surgery (Ambrosio and Tritto, 1999; de Perrot et al., 2003; Ng et al., 2006; Yellon and Hausenloy, 2007). The pathogenesis of these lung diseases may be related to lung epithelial cell damage (Matthay et al., 2012; Sweeney et al., 2013). Under normal circumstances, I/R can be understood as hypoxia-reoxygenation in tissues. However, this situation needs to be treated differently in the lungs because the alveoli contain a certain amount of oxygen, which can prevent early hypoxia caused by ischemia and maintain aerobic metabolism (de Perrot et al., 2003). Although the mechanism of lung injury induced by I/R is not completely clear, excessive production of ROS, pulmonary mitochondrial dysfunction, polymorphonuclear leukocyte infiltration, and macrophage isolation may be related to lung injury (Sommer et al., 2011; Campos et al., 2012). MG53 has been detected to be expressed in type I and type II alveolar epithelial cells, and the latter has a higher level of expression. But MG53 is shown to be not expressed in endothelial cells (Jia et al., 2014; Kim et al., 2014). In order to test whether the lack of MG53 can change the lung response after I/R-induced injury, JIA et al. conducted experiments on wild-type and MG53^−/−^ mice which induced complete left lung ischemia and hypoxia by clamping the left pulmonary artery and hilum on the procedure of 1 h ischemia/1 h reperfusion. Compared with the wild-type control, The MG53^−/−^ mouse has a lower survival rate after I/R injury. The ablation of MG53 leads to increased susceptibility of mice to I/R-induced lung injury (Jia et al., 2014), and the overexpression of MG53 has a protective effect on the repair of alveolar epithelial cells. The repair function of MG53 in the lung is related to caveolin 1(Cav1) (Kim et al., 2014). Cav1 is a protein rich in lung endothelial cells and alveolar epithelial type I cell (ATI), which promotes the repair of cell membrane rupture through endocytosis (Dahlin et al., 2004; Corrotte et al., 2013). In another animal experiment, intravenous injection of rhMG53 can have a protective effect on I/R-mediated acute lung injury (ALI) without significant toxicity. It effectively protects against lung epithelial cell damage and restores lung function after ALI (Weisleder et al., 2012; Jia et al., 2014).

### Effects of MG53 on renal I/R injury

Kidney disease is a global public health problem. With the aging of the population and the increasing incidence of diabetes and hypertension, kidney failure is also growing. Acute kidney injury (AKI) is one of the most common acute and critical illnesses in various clinical departments, with high morbidity and mortality (Doyle and Forni, 2016). Although a large number of clinical trials have been conducted with multiple interventions, a reliable method to prevent AKI has still not been found (Landoni et al., 2013). More and more scholars have realized that acute kidney injury (AKI) and chronic kidney disease (CKD) are closely related and may promote each other (Hsu and Hsu, 2016). A kidney is very sensitive to I/R, which is the most common cause of AKI. (Kanagasundaram, 2015). After renal tissue ischemia, its metabolism changes from aerobic to anaerobic, and intracellular ATP is consumed, leading to acidosis. Inactivation of Na^+^/K^+^ ATPase results in intracellular sodium and water retention, causing cell edema (Pefanis et al., 2019). Reperfusion restores aerobic metabolism in the kidney but produces reactive oxygen species (ROS), which destroy functional cells and induce the death of tubular epithelial cells (Bonventre and Yang, 2011). I/R triggers a series of harmful cellular reactions in the affected organs, ultimately leading to cell necrosis or apoptosis (Bonventre and Yang, 2011). A sign of renal I/R injury is the appearance of necrotic tubule cells (Brady and Singer, 1995).

Although MG53 is expressed at a low concentration in the kidney, it is significantly expressed in the proximal tubule epithelium (PTE). Under normal physiological conditions, the apical surface of the PTE cells has obvious endocytosis and exocytosis (Saito et al., 2010; Christensen et al., 2012). MG53-mediated membrane repair plays a vital role in renal protection. Membrane repair defects caused by MG53 deletion can increase sensitivity to I/R-induced AKI, aggravate I/R-induced AKI, and develop pathological renal phenotypes of interstitial cell damage (Duann et al., 2015). Intravenous injection of rhMG53 before I/R can effectively prevent the occurrence of AKI in the mouse model. At the same time, rhMG53 was also shown to reduce cisplatin-induced AKI without reducing its oncological efficacy. (Duann et al., 2015). Previous studies have shown that the association of rhMG53 with membrane destruction sites requires recognition of lipid signals. MG53 can bind to phosphatidylserine (PS), usually inside the plasma membrane, and may be exposed outside the plasma membrane after injury (Weisleder et al., 2012). AKI caused by I/R can lead to the exposure of phosphatidylserine on PTE cells, which may be the anchoring mechanism of the rhMG53 repair process (Duann et al., 2015). Therefore, targeting MG53 provides a new therapeutic approach to preventing I/R-related AKI.

### Effects of MG53 on hepatic I/R injury

Hepatic ischemia-reperfusion injury (HIRI) refers to the pathophysiological process in which liver ischemia is further aggravated when the blood flow is reperfused for a while. In recent years, HIRI has attracted the attention of many researchers because it often occurs in clinical settings, such as liver transplantation, liver resection, hemorrhagic shock, and trauma (Saidi and Kenari, 2014). With the development of various liver operations, HIRI has become an essential factor affecting the morbidity and mortality of the operation (Serracino-Inglott et al., 2001). As a unique and crucial immune organ of the human body, the liver contains various kinds of cells, such as Kupffer cells (KCs), natural killer cells (NK), natural killer cells (NKT), and dendritic cells (DC), which not only play an immune role but also play a pivotal role in I/R injury (Dong et al., 2007). Unlike I/R injury in other organs, there are two types of HIRI, warm IRI and cold IRI (Ikeda et al., 1992; Zhai et al., 2011). Warm IRI usually occurs when the normal blood perfusion of liver tissue is blocked, such as prolonged occlusion of blood flow during hepatectomy, shock, and trauma. Warm IRI can be divided into two distinct phases. The early stage typically occurs minutes to 6 hours after hepatic ischemia. It is characterized by the rapid activation of Kupffer cells to generate ROS, which induces oxidative stress and develops parenchymal vascular damage. The initial stage of liver injury is relatively low in extent, but it triggers a series of subsequent events. In the later stages of warm IRI, neutrophils accumulate in the liver after ischemia and directly damage hepatocytes through the production of ROS and proteases, which finally leads to cell death (Konishi and Lentsch, 2017). Cold IRI often occurs in liver transplantation because the donated liver is preserved in a hypothermic environment (Zhai et al., 2013). Two types of HIRI trigger three types of cell death: necrosis, apoptosis, and autophagy (Hu et al., 2021).

PTRF is indispensable in MG53-mediated membrane repair (Zhu et al., 2011). Although PTRF is expressed in most organs, it is not expressed in the liver (Zhou et al., 2015). Studies have shown that dysferlin is closely related to MG53 in the liver, and the C2A domain of dysferlin plays a key role (Matsuda et al., 2012). The research of Yao et al. showed that MG53 could reduce the damage of hepatocyte membrane induced by HIRI by combining with dysferlin, which could reduce the oxidative stress in HIRI and the death of hepatocytes. But the experiment failed to prove whether other specific proteins were interacting with MG53 in the liver (Yao et al., 2017). Severe liver damage caused by HIRI manifest as increased ALT and AST release. Administration of exogenous rhMG53 reduces ALT and AST in the model (Yao et al., 2017), which may be necessary for future clinical treatment of HIRI. Whether MG53 participates in the inflammatory mechanism of HIRI still needs more experiments to confirm.

## Controversy over MG53

Metabolic syndrome is not a single disease but a combination of cardiovascular disease risk factors, such as abdominal obesity, insulin resistance, hyperlipidemia, and hypertension, which increase the risk of cardiovascular atherosclerotic disease and type 2 diabetes (Kassi et al., 2011). Metabolic syndrome has become a global problem and poses a significant health risk (Saklayen, 2018). Research by Song and Wu et al. indicated that MG53 might be a pathogenic factor of diabetes (Song et al., 2013; Yi et al., 2013; Wang and Hill, 2015; Wu et al., 2019). The study by Song et al. showed that the expression of MG53 in the insulin resistance model was significantly increased, and the over-expression of MG53 triggered insulin resistance and metabolic syndrome. In contrast, ablating the MG53 did not produce the symptoms described above (Song et al., 2013). In mechanism, MG53, as an E3 ligase, performs ubiquitin-dependent degradation of insulin receptor (IR) and insulin receptor substrate (IRS1), leading to insulin resistance and metabolic disorders. (Song et al., 2013). Wu et al. demonstrated that high glucose or high insulin induced MG53 secretion in isolated rodent hearts and skeletal muscles. In humans and rodents with diabetes, increased glucose was accompanied by increased circulating MG53 (Wu et al., 2019). Mechanistically, MG53 binds to the extracellular structural domain of the insulin receptor and inhibits insulin signaling (Wu et al., 2019). Liu et al. proposed different mechanisms of MG53-mediated diabetes. They found that overexpression of MG53 was sufficient to induce systemic insulin resistance and impaired glucose uptake by constructing MG53-overexpressed mice using an α -myosin heavy chain (α-MHC) promoter (Liu et al., 2015a). Mechanistically, in addition to MG53-induced dysregulation of IR and IRS-1, it also blocks insulin signaling by upregulating peroxisome proliferator-activated receptor-α (PPAR-α) levels, resulting in cardiac lipid accumulation and, ultimately, diabetic cardiomyopathy (Finck et al., 2003; Liu et al., 2015a).

However, another study differs from previous results; compared with wild-type (WT) littermates, the circulating MG53 in blood samples from diabetic mice was significantly reduced (Wang et al., 2020). Wang et al. established two db/db mouse models, one of which removed MG53 from the blood, and the other continuously increased MG53 in the blood. As a result, the insulin signal and glucose treatment remained unchanged. Treatment of MG53^−/−^ mice with streptozotocin (STZ) leads to abnormal glucose therapy, which indicates that MG53 might have protective *ß* -cell function (Wang et al., 2020). The evidence provided by Bian et al. shows that the continuously rising circulating MG53 did not affect insulin signal and glucose processing in mice, and it is safe for metabolism and heart function (Bian et al., 2019). Research by Philouze et al. showed that MG 53 was not a key regulator of skeletal muscle insulin signaling pathway, and these findings were consistent with the results of BIAN et al. (Philouze et al., 2021). Ma et al. established a mouse model of metabolic syndrome to investigate the effect on the activity of MG53. They found that the level of circulating MG53 in mice fed with a high-fat diet decreased significantly, but the expression of MG 53 in skeletal muscle and myocardium remained unchanged (Ma et al., 2015). The differences in these results have led to controversy over MG53, which will require more studies to resolve.

## The therapeutic potential of recombinant human MG53

To find out whether rhMG53 can play a therapeutic role in heart IRI, Liu et al. demonstrated by using several animal models that administration of rhMG53 reduced infarct size after reperfusion (Liu et al., 2015b). Wang et al. used rhMG53 to treat alkali-induced corneal wounds and found that topical application of rhMG53 significantly improved corneal wound healing in diabetic mice. Compared with WT mice, the cornea of db/db mice undergoes excessive revascularization after alkaline injury, and the addition of rhMG53 alleviates the excessive vascularization (Wang et al., 2020). Sepsis will lead to the down-regulation of MG53 and PPARα. Supplementation of rhMG53 can improve the survival rate and cardiac function, reduce oxidative stress, diminish inflammation and decrease cardiomyocyte apoptosis related to PPARα up-regulation (Han et al., 2020). MG53 supplements can likewise protect the heart from myocardial dysfunction caused by sepsis by up-regulating PPARα expression (Han et al., 2020). As previously mentioned, multiple articles support the value of rhMG53 in therapy, but the safety of rhMG53 needs to be further assessed before its application. Repeated intravenous administration of rhMG53 in rodents and dogs did not produce adverse effects or alter rat blood metabolites (Weisleder et al., 2012; Duann et al., 2015; Wang et al., 2020). These results will help its potential clinical application.

## Conclusion

**FIGURE 2:**
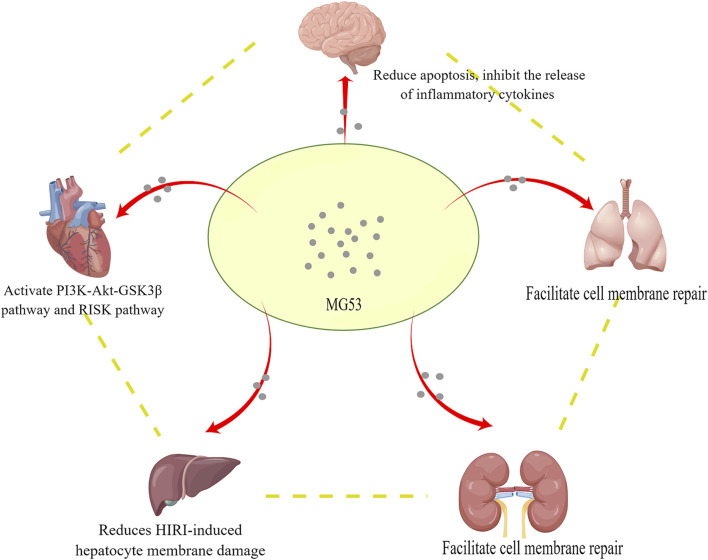
MG53 protects multiple organs from ischemia-reperfusion injury through blood circulation.

Since I/R injury is a complex pathophysiological process, it can affect multiple organs, such as the heart, brain, lung, kidney, and liver. ATP depletion, ROS production, and elevated intracellular and mitochondrial calcium (calcium overload) are all related to I/R, and their clinical manifestations are diverse, which poses a severe challenge for clinicians. MG53 is mainly expressed in skeletal muscle and myocardium and also plays a unique role in other organs (Figure 2), for example, in promoting the repair of the cell membrane and activating the cardiac RISK pathway to mediate cardiac IPC and PostC. Currently, almost all studies on the function of MG53 are conducted in animal models. Further work is to conduct clinically relevant studies. Although the mechanism related to I/R injury still needs to be developed, we believe that MG53 provides new ideas for exploring the treatment of I/R injury.
